# Changes in Phenolic Composition and Bioactivities of Ayocote Beans under Boiling (*Phaseolus coccineus* L.)

**DOI:** 10.3390/molecules29163744

**Published:** 2024-08-07

**Authors:** Ramiro Baeza-Jiménez, Leticia X. López-Martínez

**Affiliations:** 1 Laboratorio de Biotecnología y Bioingeniería, Centro de Investigación en Alimentación y Desarrollo, A.C. Av. Cuarta Sur 3820, Fracc. Vencedores del Desierto, Delicias CP 3089, Chihuahua, Mexico; ramiro.baeza@ciad.mx; 2 Laboratorio de Antioxidantes y Alimentos Funcionales, Centro de Investigación en Alimentación y Desarrollo, CONAHCYT-CIAD, A.C. Carr. Gustavo Enrique Astiazarán Rosas 46, Col. La Victoria, Hermosillo CP 83304, Sonora, Mexico

**Keywords:** *Phaseolus coccineus*, phenolic compounds, bioactivities, thermal treatment, HPLC-MS

## Abstract

Ayocote beans (*Phaseolus coccineus* L.) are a rich source of some bioactive molecules, such as phenolic compounds that exhibit antioxidant capacity that promote health benefits. Ayocote is mainly consumed after cooking, which can impact the antioxidant characteristics of the phenolic compounds responsible for some of its health benefits. Therefore, this study investigated the effects of boiling on the phenolic composition and bioactivities of ayocote beans before and after boiling. Boiling decreased the total phenolic content (70.2, 60.3, and 58.2%), total anthocyanin (74.3, 80.6, and 85.7%), and antioxidant activity (DPPH: 41.2, 46.9, and 59.1%; ORAC: 48.23, 53.6 and 65.7%) of brown, black, and purple ayocote beans, respectively. All the extracts also inhibited the activity of α-glucosidase with efficacy values from 29.7 to 87.6% and α-amylase from 25.31 to 56.2%, with moderate antiglycation potential (15.2 to 73.2%). Phenolic acids, anthocyanins, and flavonoid decreases were detected in boiled samples by HPLC-MS analysis. Although boiling reduced the phenolic compounds, bioactive compounds remained in a considerable content in boiled ayocote.

## 1. Introduction

Of the 52 species of the *Phaseolus* genus, the ayocote (*Phaseolus coccineus* L.) is poorly studied. Ayocote is not considered in the official statistics, because its cultivated area and total production are included in those of common beans, although it is the third most economically important after *P. vulgaris* L. and *P. lunatus* L. Rawal [1]. Ayocote is a pulse with a high content of protein and is rich in phytochemicals, such as phenolic compounds, which possess activities beneficial to health, such as antioxidant, antiobesogenic, antihypertensive, anticancer, and postprandial hyperglycemia control [2,3,4]. Before consumption, thermal processing is applied to pulses to achieve desirable sensory characteristics; however, thermal processing may also cause a significant impact on bioactive compounds [5], producing changes in antioxidant activity and bioactive characteristics [6], such as the potential to inhibit digestive enzymes involved in the intestinal digestion of carbohydrates α-amylase and α-glucosidase and the inhibition of glycation of proteins—bioactivities implicated in the pathogenesis of diabetes [7]. The inhibition of α-amylase and α-glucosidases delays the digestion and absorption of carbohydrates, thus suppressing postprandial hyperglycemia [8]. Advanced glycation end products (AGEs) are one of the significant pathogenic events in diabetic problems, including retinopathy and cataracts [9]. Thus, investigating α-amylase, α-glucosidase, and AGE inhibitors would offer a possible tactic for preventing diabetic complications.

Open cooking or boiling is the most common method of cooking beans [10]; nevertheless, the loss of water-soluble phenolic compounds is possible in this treatment due to leaching during soaking and boiling [11]. Moreover, high temperatures can generate oxidative degradation, the breakdown of phenolic structure, and the formation of a complex of phenolic compounds with anthocyanins, tannins, and proteins, reducing their extractability [12]. Therefore, this work aimed to characterize the phenolic content, in vitro antioxidant, and antidiabetic activities of ayocote beans before and after boiling. The results of this study will provide valuable evidence on the phenolic compound composition of cooked ayocote beans to promote their consumption and their relationship with the reduction in disease risk.

## 2. Results and Discussion

Table 1 depicts the total phenolic content, total anthocyanin, and antioxidant activity from the raw and boiled black, purple, and brown ayocote beans. Statistical differences (*p* < 0.05) were observed among the varieties in both forms.

### 2.1. Total Phenolic Content (TPC)

The data show that TPC ranged from 152.76 to 263.28 mg GAE/100 g for raw samples (Table 1). The determined TPC overlapped with those stated by Alvarado-López et al. [2], ranging from 156.2 to 207.0 mg/100 g in pigmented ayocote bean varieties from different states in Mexico. Nevertheless, the values are higher than those reported by García-Alonso et al. [13] in purple and black ayocote, varying from 669 to 673 mg GAE/100 g and 59.3 mg GAE/100 g in black ayocote reported by Osuna-Gallardo et al. [14]. The differences in TPC on ayocote between studies could be attributed to the variety studied, agronomic practices, maturity at harvest, storage conditions, and the solvents used during extraction. When thermal treatment was applied, TPC was significantly (*p* < 0.05) reduced compared to raw beans (Table 1). During boiling, there are more significant losses on the brown variety (70.2%), followed by black (60.3%) and purple (58.2%) varieties. In a different study, García-Alonso et al. [13] reported losses of 77 and 83.2% of TPC in purple and black ayocote beans cooked in boiling water at atmospheric pressure (98 °C) for 60 min; on the other hand, Corzo-Ríos et al. [15] reported that purple and brown runner beans (*P. coccineus*) cooked in an autoclave (15 lb/in^2^ at 120 °C for 20 min) underwent a reduction of 25.0 and 33.5% of TPC, respectively. The treatment in which the beans were cooked and the processing time can explain their results and our findings. Cooking promotes the rupture of the cell wall of the ayocotes, causing fluid migration from the cell to the water extracellular medium, extracting soluble compounds such as TPC [16].

### 2.2. Total Anthocyanins Content (TAC)

TAC in raw ayocote beans ranged from 21.42 to 86.92 mg C3GE/100 g, where the purple ayocote beans attained the highest concentration (Table 1); these values were higher than those reported by Quiróz-Sodi et al. [17] in three types of ayocote beans from Mexico (from 4.40 to 12.20 mg C3GE/100 g). Furthermore, Capistrán-Carabarin et al. [18] reported higher values from 137 to 150 mg C3GE/100 g in pigmented ayocote beans: boiling reduced the TAC concentration by 74.3, 80.6, and 85.7% for brown, black, and purple ayocotes, respectively (Table 1). Since anthocyanins are highly water-soluble, treatments using water such as soaking or boiling will lead to anthocyanin leaching [11], besides their interaction with other compounds, and mainly the decomposition or degradation for thermal treatments caused losses of anthocyanins [19].

### 2.3. Antioxidant Activity

The varieties exhibiting the greatest DPPH-reducing capacity were those that presented the highest number of anthocyanins (Table 1). These results are consistent with Alvarado-López et al. [2], who reported that varieties of *P. coccineus* L. with dark-colored testa (rich in anthocyanins) showed the highest antioxidant activity. Compared to the DPPH values reported in the same study, the authors reported values from 1420 to 1740 µmol TE/100 g, results that overlapped with our values but were higher than those reported by García-Alonso et al. [13], which ranged from 834 to 838 µmol TE/100 g in different varieties of ayocote beans. The variations in DPPH values are possibly related to the specific composition of anthocyanin derivatives in the ayocotes in each study, such as simple or acylated glycosides of cyanidin, pelargonidin, or peonidin [20]. The antioxidant activity determined by DPPH decreased by 59.1% in purple, 46.9% in black, and 41.2% in brown boiled ayocote beans (Table 1). García-Alonso et al. [13] reported that black and purple ayocote beans underwent about a 30% reduction in antioxidant activity analyzed by DPPH after cooking.

The loss of DPPH free radical scavenging capacity could be partly due to the loss of soluble antioxidants such as flavonoids and anthocyanins when soaking in water. In addition, the heating effect could cause partial alterations in the structure of the phenolic compounds [21], besides the formation of complexes of phenolic compounds with proteins and carbohydrates, which makes them unavailable for exerting their antioxidant capacity [22].

According to our findings (Table 1), the highest ORAC value was reached for raw purple ayocote beans (6162.65 µmol TE/100 g), followed by black (4234.29 µmol TE/100 g) and brown (3096.62 µmol TE/100 g) ayocote beans (Table 1). Our values are lower than those previously reported by Xu and Chang [21] for black beans (7927 µmol TE/100 g). Ayocote beans showed higher ORAC values than those reported by Osuna-Gallardo [14] in black ayocote beans from Mexico, showing ORAC values of 3866.37 µmol TE/100 g. Overall, the cooking treatment caused significant (*p* < 0.05) decreases in ORAC (48.23 to 65.71%) compared to raw ayocote beans. Similar behavior was proved by Fuentes et al. [23] in beans (*P. vulgaris* L.) subjected to boiling; the authors described losses of antioxidant activity from 40 to 60%, conversely to what was reported by Osuna-Gallardo et al. [14], who determined values of 25% in black ayocote beans. The cooking treatment significantly (*p* < 0.05) reduced the antioxidant capacity of the ayocote beans, as indicated by a decrease in the DPPH and ORAC values; even though boiled ayocote beans underwent a greater extent of phenolic compound degradation, they retained their antioxidant activity.

### 2.4. α-Amylase and α-Glucosidase Inhibition

Delaying intestinal glucose absorption can decrease glucose uptake, and minimizing glycemic spikes to maintain blood glucose within normal levels is a promising strategy for controlling and delaying the early onset of diabetes-associated complications [24]. The use of inhibitors from natural sources, such as phenolic compounds, has been shown to be a potential inhibitor of α-amylase and α-glucosidase enzymes implicated in the intestinal digestion of carbohydrates [8,25].

**Figure 1:**
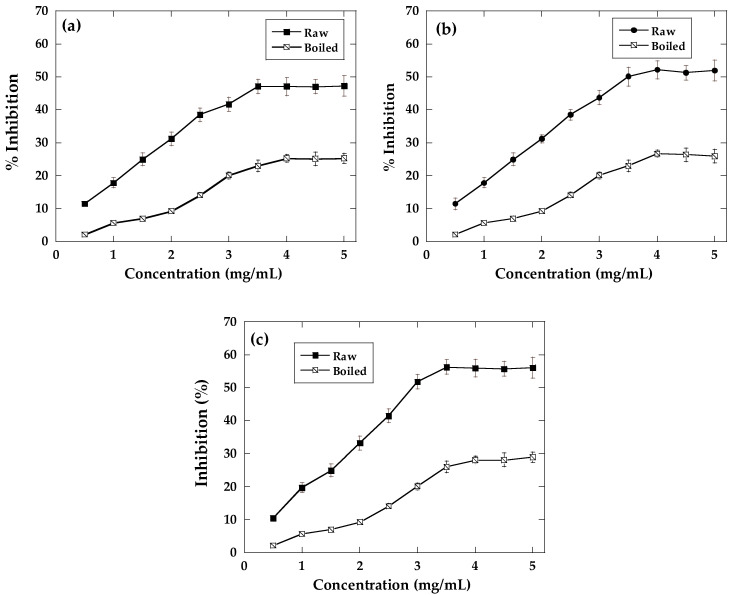
α-amylase inhibition of (**a**) brown, (**b**) black, and (**c**) purple raw and boiled ayocote beans. Values are presented as mean ± standard deviation (*n* = 3). Different letters indicate significant differences (*p* < 0.05).

**Figure 2:**
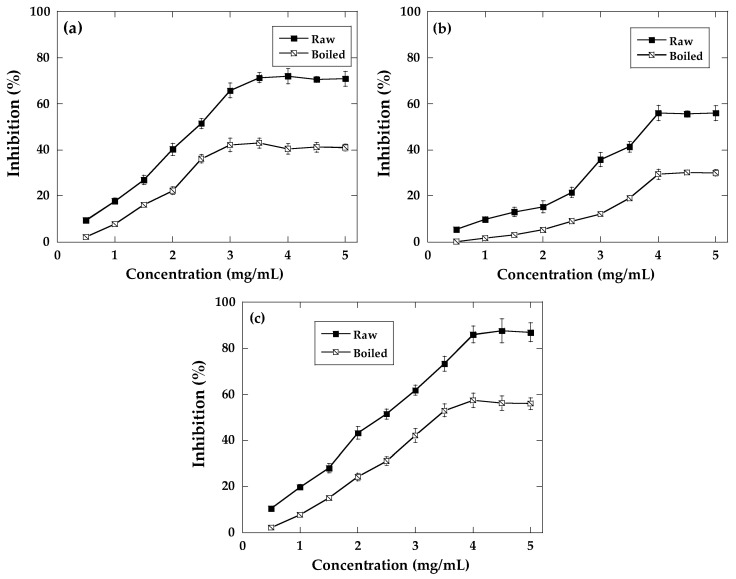
α-glucosidase inhibition of (**a**) brown, (**b**) black, and (**c**) purple raw and boiled ayocote beans. Values are presented as mean ± standard deviation (*n* = 3).

All ayocote bean extracts could inhibit α-amylase and α-glucosidase in a dose-dependent manner before and after boiling (Figure 1 and Figure 2). The inhibitory activities of α-amylase were moderate in raw and boiled beans without significant differences (*p* > 0.05). The inhibitory capacity decayed with thermal processing in the three varieties. The extracts of the purple variety showed inhibition (raw and boiled; 52.3 and 28.1%, respectively), followed by black (50.1 and 26.7%) and brown (50.2 and 25.3%) varieties at the maximum dose (7.0 mg/mL) (Figure 1). The presence of phenolic acids such as ferulic and gallic acid, quercetin, and myricetin and anthocyanins such as cyanidin 3-glucoside in ayocote bean extracts, identified in ayocote bean extracts (Table 2) which are well-known α-amylase inhibitors, could be a significant factor that determines the inhibitory capacity [26,27].

In the case of α-glucosidase, the lowest inhibition was for black variety extracts (55.8%) and the highest for purple variety (87.6%). Figure 2 displays the effect of boiling on the α-glucosidase inhibitory activities of ayocote bean extracts. A significant decrease in the α-glucosidase inhibition was observed in the three varieties after boiled treatment.

Despite phenolic acids and anthocyanins from common beans showing the capacity to inhibit enzymes involved in carbohydrate metabolism and decrease the availability of α-glucosidase, the results observed suggest that non-phenolic compounds, including peptides, may be involved in the inhibition [28]; similar behavior was reported by [28,29] in different cultivars of common beans (*P. vulgaris*).

Although the inhibitor effects were lower than those of acarbose (89.6%), the results indicated that the extracts of ayocote beans could be suitable inhibitors of α-amylase and α-glucosidase activities and can inhibit these enzymes with minimum side-effects [30]. Scarce research has been conducted on the inhibition of α-amylase and α-glucosidase for extracts of ayocote beans.

Studies have highlighted the potential of phenolic compounds as potent α-glucosidase inhibitors and moderate α-amylase inhibitors, which could avoid the side-effects of synthetic drugs, such as gastrointestinal side-effects [31]. The results of this study show similar behavior and a greater capacity to inhibit α-glucosidase activity compared to α-amylase, making them promising candidates to be used as an alternative to synthetic molecules or to complement them.

### 2.5. AGE Inhibition

**Figure 3:**
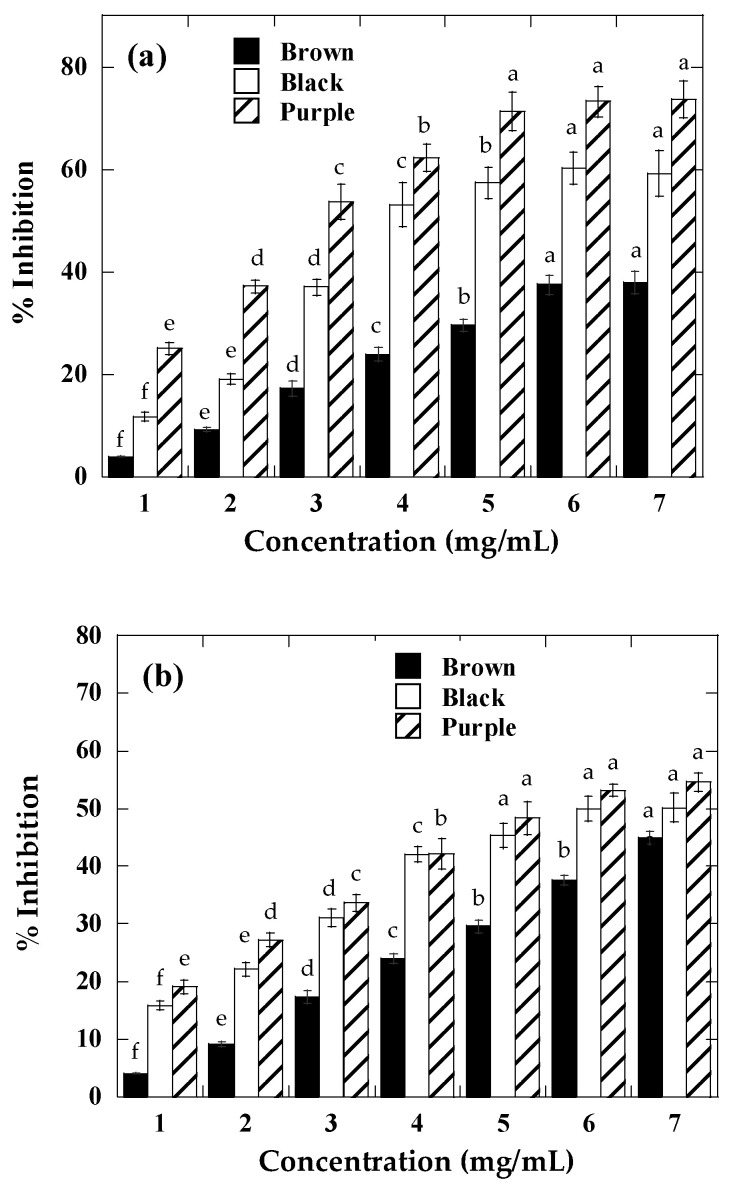
Antiglycation activity of (**a**) raw and (**b**) boiled ayocote beans. Values are presented as mean ± standard deviation (*n* = 3). Different letters indicate significant differences (*p* < 0.05).

AGEs are formed irreversibly during protein glycation and are directly linked to several diseases, such as diabetes [32]. It has been shown that extracts rich in phenolic compounds can inhibit AGE formation [25]. Thus, investigating natural compounds that can prevent glycation could be a therapeutic option to inhibit the complications associated with diabetes [33]. However, studies on phenolic compounds present in pulses such as ayocote bean have been little explored. The analyzed extracts of raw and boiled ayocote showed a dose-dependent antiglycation activity: for raw ayocote, IC_50_ = 3.27 mg/mL and 4.43 mg/mL, with non-detected values and an inhibition potential of AGE formation of 73.2, 57.2, and 37.15% at the maximum concentration tested (7.0 mg/mL) for purple, black, and brown extracts, respectively (Figure 3a). The antiglycation activity correlated positively with the total phenolic and anthocyanin content, proving that phenolic compounds can act as inhibitors of BSA glycation [34]. Ferulic acid, gallic acid, cyanidin 3-glucoside, and malvidin 3-glucoside compounds present in ayocote bean extracts (Table 2) have been reported capable of inhibiting the advanced phase of glycation and decreasing protein carbonyl and AGE formation [35,36]. After thermal treatment, the extracts displayed weaker activities, with IC_50_ = 6.2 mg/mL and 6.4 mg/mL, and non-detected values in purple, black, and brown boiled ayocote beans. The inhibition potential of AGE formation varied from 15.2 to 53.1% (Figure 3b). This decrease in activity may be directly related to the decrease in phenolic acids, flavonoids, and anthocyanins (Table 2).

Aminoguanidine is a synthetic glycation inhibitor whose value of antiglycation showed in this study had an IC_50_ of 0.87 mg/mL and an inhibition potential of AGE formation of 87.3% at 6.5 mg/mL. Our report, for the first time, determined the antiglycation capacity of ayocote bean extract. The results suggest that ayocote beans are bioactive compounds that may be beneficial in controlling or preventing AGE generation; however, their efficacy in vivo must still be meticulously investigated.

### 2.6. Phenolic Profile

The phenolic acid composition of raw and boiled brown, purple, black, and ayocote beans is shown in Table 2.

The reduction in the gallic acid content on boiled ayocote beans ranged from 67.5 to 83.1%, while the sinapic acid decreased from 84.7 to 91.2%. The losses of ferulic acid varied from 84.76 to 91.2%, the reduction in chlorogenic acid ranged from 66.8 to 72.3%, and the protocatechuic acid content was completely lost (Table 2). In agreement with our results, Xu and Chang et al. [22] reported that boiling decreased the gallic acid content by 50.1% in black beans (*P. vulgaris* L.). Conversely, Teixeira-Guedes et al. [37] reported that boiling increased gallic acid from 1.40- to 3.47-fold, chlorogenic acid from 1.56- to 2.51-fold, p-coumaric acid from 1.44- to 2.51-fold, and ferulic acid approximately two times in kidney, pinto, black, and borlotti beans (*P. vulgaris* L.).

Overall, gallic, sinapic, ferulic, chlorogenic, p-coumaric, and protocatechuic acids were detected in the three varieties of raw ayocote beans, with chlorogenic acid being the most predominant in the following order: purple > black > brown. 4-Hydroxybenzoic acid was only identified in black ayocote beans and boiling significantly (*p* < 0.05) decreased individual phenolic acids (Table 2).

The decrease in phenolic acids upon cooking may primarily be a consequence of oxidative degradation (including enzymatic browning) and the formation of phenolic complexes with proteins, anthocyanins, and tannins [12]. The structure of phenolic acids could also be affected by other factors; for example, the remarkable reduction in the chlorogenic acid content reveals the high susceptibility of their esterified bonds to heat [38].

The technical literature related to ayocote beans in terms of their flavonoid composition is limited. In this regard, the presence of catechin, apigenin, and kaempferol in three types of pigmented ayocote beans from Mexico has been reported [17]. Our results show the presence of kaempferol 3-glucoside, catechin, and epicatechin in the three varieties analyzed; myricetin and quercetin were detected only in raw black ayocote (Table 2). Regular boiling significantly (*p* < 0.05) decreased the kaempferol 3-glucoside content by 100% for black, 97.3% for purple, and 95.8% for brown ayocote beans.

The catechin content decreased by 63.1%, while epicatechin was reduced by 66.3% in black beans and was completely lost in purple and brown ayocote beans (Table 2). By contrast, Ranilla et al. [39], using Brazilian beans (*P. vulgaris* L.), reported an increase in kaempferol from 307.2 to 488.0 µg/g and in quercetin from 7.8 to 13.3 µg/g after cooking. After boiling, the significant loss of flavonoids may be due to the combined effects of soaking and thermal degradation [40]. In particular, catechin and epicatechin decreased significantly due to their highly hydroxylated structures, which are vulnerable to redox reactions, causing losses when beans are subjected to heat treatment. Moreover, at elevated temperatures, catechin and epicatechin are susceptible to polymerization and non-enzymatic condensation. Kaempferol, myricetin, and quercetin are reported to undergo decarboxylation, resulting in the polymerization of phenols, reducing their extractability at high temperatures, and the breakdown of phenolic structures [12] may be some factor in the decrease in these compounds during cooking.

Studies regarding anthocyanins in ayocote beans are scarce. In this sense, Yoshida et al. [41] reported the presence of cyanidin 3-glucoside and delphinidin 3-glucoside in three different varieties of light red runner beans (*P. coccineus* L.) from Japan; the same anthocyanins are described in our results (Table 2).

Six anthocyanins, namely delphinidin 3-glucoside, cyanidin 3-glucoside, petunidin 3-glucoside, malvidin 3,5-diglucoside, malvidin 3-galactoside, and malvidin 3-glucoside, were detected in brown, purple, and black raw ayocotes. The dominant components were delphinidin 3-glucoside, malvidin 3-glucoside, and petunidin 3-glucoside (Table 2). These findings are in accordance with those of Xu and Huang [42], who reported that those anthocyanins are the major anthocyanins in common black beans.

Boiling significantly (*p* < 0.05) reduced the content of each anthocyanin in ayocote beans, causing losses of 100, 97.2, and 97.8% of delphinidin 3-glucoside; 100, 88.5, and 97.8% of cyanidin 3-glucoside; 95.6, 98.2, and 92.3% of petunidin 3-glucoside; 88.0, 72.1, and 74% of malvidin 3,5-diglucoside; and 91, 91.9, and 91.4% of malvidin 3-glucoside in brown, purple, and black varieties, respectively. Malvidin 3-galactoside was not detectable in any of the cooked varieties.

Previous studies have reported that the number, location, and nature of sugars attached to the aglycone are closely associated with the stability of anthocyanins. For example, non-acylated anthocyanins are more unstable than acylated ones [43]. Based on the current data (Table 2), ayocote beans contained predominantly non-acylated simple anthocyanins, which may be responsible for the higher degradation ratio in the present study; furthermore, the stability of anthocyanins is inversely related to temperature [11]. Moreover, Dumitraşcu et al. [44] suggested that the thermal degradation of anthocyanins might be partially explained by intramolecular co-pigmentation and intermolecular self-association reactions. In addition, it is essential to point out that draining the soaking water decreases the levels of the different phenolic compounds and the effects described above.

## 3. Materials and Methods

### 3.1. Plant Material

Three varieties of ayocote beans (*Phaseolus coccineus* L.) were evaluated in the present study. The varieties were collected from different regions of Mexico in 2017: a purple variety was obtained from San Antonio Tecajetes, Puebla (18°57′37.80″ N and 97°51′21.48″ W); a black variety was collected in Chalco, Estado de México (19°15′1.86″ N and 98°53′47.60″ W); and a brown variety was cultivated in Nealtican, Puebla (19°2′53.79″ N and 98°25′36.98″ W).

### 3.2. Boiling Treatment

Twenty-five grams of seeds of each genotype of ayocote beans were soaked in 250 mL of distilled water for 10 h; after this time, the water was drained. Then, samples were subjected to atmospheric boiling (98 °C, for 75 min) at a sample:/water ratio of 1:10 (*w*/*v*). Ayocote beans are considered cooked when they yield easily to pressure between the thumb and index finger and present a soft, paste-like consistency that ranges from fine to slightly lumpy. After boiling, the ayocote beans and the water cooking were pooled, cooled at room temperature, frozen at −18 °C, and then lyophilized in a freeze-drier (Virtis Research Equipment, Gardiner, NY, USA). The lyophilized seeds were stored at 4 °C in sealed plastic bags until further analysis.

### 3.3. Extract Preparation

The extracts were obtained according to Fontes-Zepeda et al. [45] with some modifications. Briefly, raw and lyophilized cooked ayocote were ground to a fine flour with an IKA all basic mill (IKA Works, Inc., Wilmington, NC, USA) to pass through a 40-mesh sieve. A sample (2.5 g) of flour was homogenized with 10 mL of a mixture of ethanol/water (80:20 *v*/*v*) using an Ultra Turrax T25 basic homogenizer (IKA Works, Wilmington, NC, USA). The homogenate was sonicated in a Bransonic 2210 sonicator (Bransonic Ultrasonic Co., Danbury, CT, USA) for 30 min and then centrifuged at 6000× *g* in a centrifuge (Beckman Coulter, AllegraTM 64R, Brea, CA, USA) for 10 min at 4 °C. The supernatant was pooled, and the precipitate was extracted again under the abovementioned conditions. Ethanol was removed by rotary evaporation, and the remaining water was removed by freeze-drying. The dry ayocote bean extracts were stored in amber vials for further analysis. The dry extract was dissolved in 5 mL of 80% ethanol and used to quantify total phenolic content and antioxidant activity.

### 3.4. Phenolic Content Assays

#### 3.4.1. Determination of Total Phenolic Compounds (TPC)

The TPC of the extracts was analyzed according to Swain and Hillis [46], with some modifications. Briefly, 15 μL of ethanolic extracts was mixed with 240 μL of distilled water and 15 μL of 2 N Folin–Ciocalteu reagent in a 96-well Costar^®^ flat-bottom microplate. After incubation for 3 min, 30 μL of 4 N Na_2_CO_3_ was added to the plates and then allowed to stand in the dark for 90 min. The absorbance was measured at 725 nm using a Synergy HT Absorbance Microplate Reader (BioTek Co., Winnoski, VT, USA). A standard calibration curve was prepared using gallic acid, and the TPC was calculated and is expressed as mg of gallic acid equivalents per 100 g of dry weight (mg GAE/100 g dw).

#### 3.4.2. Determination of Total Anthocyanin Compounds (TAC)

This analysis was carried out according to Abdel-Aal and Hucl [47]. Briefly, 3 g of sample was diluted with 10 mL of a cold acidified ethanolic solution (ethanol and 1 N HCl, 85:15 *v*/*v*, pH 1.0) with constant stirring for 30 min and centrifuged at 16,600× *g* for 15 min, and the supernatant was collected. The absorbance was measured at 535 and 700 nm using a microplate reader. TAC was calculated as follows:

C = [(Abs_535_ − Abs_700_)/ε] × VT × MW × (1/weight of sample dw)

where C is the total anthocyanin concentration expressed as mg of cyanidin 3-glucoside equivalents per 100 g (C3GE/100 g), ε is a molar absorptivity of 25,965 M^−1^ cm^−1^, MW is a molecular weight of 449.2 g mol^−1^, and VT is the total volume of extract.

### 3.5. Antioxidant Capacity Assays

The free-radical-scavenging activity of the extracts was measured using DPPH (2,2-diphenyl-1-picril-hydracyl) radicals [48]. A Trolox (6-hydroxy-2,5,7,8-tetramethylchroman-2-carboxylic acid) curve from 0.05 to 1 µmol TE/g was used to calculate the concentration, which is expressed as µmol of Trolox equivalents per 100 g (µmol TE/100 g), and the ORAC (oxygen radical absorbance capacity) assay was performed using fluorescein as the fluorescent probe, AAPH [2,2-azobis (2-amidino-propane] dihydrochloride] as a peroxyl radical generator, and Trolox as a standard [49]. A Trolox curve from 6.25 to 125 µmol TE/100 g was used to calculate the results, which are expressed as µmol of Trolox equivalents per 100 g of dry weight (µmol TE/100 g dw).

### 3.6. α-Amylase Inhibition

α-amylase was determined according to the Worthington Enzyme Manual [50] with some modifications. Briefly, 500 µL of a serial dilution (1 to 10 mg/mL) of the dry extract was prepared in phosphate buffer (0.2 M, pH 6.8), or positive control (1 mM acarbose) was added to 500 µL of 13 U/mL a-amylase solution (0.02 M sodium phosphate buffer, pH 6.9) and incubated in test tubes at 25 °C for 10 min before 500 µL of 1% soluble starch solution (dissolved in sodium phosphate buffer and boiled for 15 min) was added to each tube and incubated for another 10 min. Finally, 1 mL of dinitrosalicylic acid color reagent was added, and the tubes were placed in a 100 °C water bath for 5 min. The mixture was diluted with 10 mL of distilled water; after that, the absorbance was read at 520 nm, and the inhibitory effects of the extracts on α-amylase were calculated and are expressed as percentage (%).

### 3.7. α-Glucosidase Inhibition

The α-glucosidase assay was performed according to Cuevas-Juárez et al. [51] in a 96-well plate, and 50 µL of extracts at different concentrations (1 to 10 mg/mL) was prepared in phosphate buffer (0.2 M, pH 6.8) or positive control (1 mM acarbose) was added to 100 µL of a 1 U/mL α-glucosidase solution (in 0.1 M sodium phosphate buffer pH 6.9) and incubated for 10 min. A 50 µL aliquot of a 5 mM p-nitrophenyl-a-D glucopyranoside solution (in 0.1 M sodium phosphate buffer pH 6.9) was added briefly to each well and incubated at 25 °C for 5 min. The absorbance was read at 405 nm. Absorbances were used to calculate the inhibitory effects of extracts on α-glucosidase, and results are expressed as percentage (%).

### 3.8. Antigycation Inhibition Activity

A bovine serum albumin (BSA)-glucose model was used to evaluate the inhibition effect of the extracts on the formation of AGEs [52]. Briefly, 2.5 g of BSA and 7.2 g of D-glucose were dissolved in 1.5 M phosphate buffer (pH 7.4) to obtain the control solution with 50 mg/mL of BSA and 0.8 M D-glucose. Two milliliters of the control solution was incubated at 37 °C for 7 days in the presence or absence of 1 mL of ayocote bean extracts (in a 1.5 M phosphate buffer, pH 7.4). After 7 days of incubation, fluorescent intensity (excitation at 330 nm and emission at 410 nm) was measured, and the results are calculated and expressed as the half-maximal inhibitory concentration (IC_50_ in mg/mL).

### 3.9. HPLC-DAD and ESI-MSD Conditions

The extracts were analyzed by high-performance liquid chromatography (HPLC; Agilent model 110 system, Wilmington, DE, USA), coupled with a photodiode array detector (DAD), according to Lin et al. [53] with modifications by López-Martínez et al. [54]. Phenolic compounds were identified by matching their spectral (UV-Vis or MS) characteristics against standards or derived from published data. All standards were purchased from Sigma-Aldrich (Steinheim, Germany).

### 3.10. Statistical Analysis

All experiments were performed in triplicate and are expressed as the mean (*n* = 3) ± standard deviation. Obtained data were analyzed by PROC ANOVA, and differences among them were evaluated by a Tukey test (*p* < 0.05) using SAS 9.0.

## 4. Conclusions

We conducted this study to encourage the consumption and revalorization of the underutilized pulse ayocote (*P. coccineus* L.), which possesses bioactive compounds with preventive effects against some chronic diseases. Phenolic compounds in purple ayocote beans demonstrated a higher antioxidant activity against DPPH and ORAC and showed the highest content of phenolic compounds and individual anthocyanins. The phenolic concentration varied among varieties, but this did not affect the potential benefits of their bioactive content. Boiling significantly affected the TPC, TAC, antioxidant activity, individual phenolic compounds, and the bioactivities studied in the three varieties of ayocote beans. However, a considerable antioxidant activity and inhibition capacity remain in boiled ayocote beans. Thus, thermal treatment does not fully modify the functional properties of the beans, whereby ayocotes could be an important functional food or used as a bioactive ingredient for the preparation of new foodstuffs. More studies must be directed to preserve the biodiversity of Mexican beans, including ayocote beans, which are potentially exploitable as a staple.

## Figures and Tables

**Table 1:** Effect of boiling on phenolic compounds and antioxidant activities of purple, black, and brown ayocote beans.

Sample	TPC (mg GAE/100 g dw)	TAC (mg C3GE/100 g dw)	DPPH (µmol TE/100 g dw)	ORAC (µmol TE/100 g dw)
Brown ayocote				
Raw	152.73 ± 10.19 ^cA^	21.42 ± 0.98 ^cA^	1630.27 ± 22.11 ^cA^	3096.62 ± 20.16 ^cA^
Cooked	45.49 ± 1.25 ^cB^	5.50 ± 0.34 ^bB^	958.44 ± 19.36 ^bB^	1061.61 ± 26.15 ^bB^
Black ayocote				
Raw	218.29 ± 7.56 ^bB^	75.47 ± 4.16 ^bA^	1976.09 ± 16.97 ^bA^	4234.29 ± 10.48 ^bA^
Cooked	86.87 ± 3.33 ^bA^	14.61 ± 0.05 ^aB^	1049.13 ± 9.44 ^aB^	2049.26 ± 21.33 ^aB^
Purple ayocote				
Raw	263.20 ± 9.25 ^aA^	86.92 ± 4.25 ^aA^	2022.51 ± 23.16 ^aA^	6162.65 ± 14.52 ^aA^
Cooked	110.17 ± 6.19 ^aB^	12.43 ± 1.19 ^aB^	827.01 ± 10.28 ^cB^	2113.56 ± 16.25 ^aB^

Data are expressed as mean ± standard deviations (*n* = 3). Different letters indicate significant differences (*p* < 0.05). Lowercase letters indicate differences among beans of varieties (*p* < 0.05), and capital letters indicate differences between treatments. TPC: total phenolic content, TAC: total anthocyanins content, DPPH: 2,2-diphenyl-1-picril-hydracyl, ORAC: the oxygen radical absorbance capacity, dw: dry weight.

**Table 2:** Effect of boiling in the phenolic profile of ayocote beans (µg/g dw).

Ayocote Beans						
	Black		Purple		Brown	
Phenolic acids						
**Compounds**	**Raw**	**Boiled**	**Raw**	**Boiled**	**Raw**	**Boiled**
**GA**	8.46 ± 0.81 ^a^	3.32 ± 1.23 ^b^	40.21 ± 6.61 ^a^	7.46 ± 0.36 ^b^	60.27 ± 1.61 ^a^	10.17 ± 0.97 ^b^
**SA**	20.12 ± 1.56 ^a^	4.12 ± 1.16 ^b^	40.46 ± 4.55 ^a^	5.29 ± 0.32 ^b^	60.03 ± 3.41 ^a^	5.23 ± 0.51 ^b^
**FA**	21.06 ± 1.64 ^a^	0.27 ± 0.11 ^b^	130.38 ± 5.80 ^a^	1.42 ± 0.37 ^b^	80.45 ± 5.11 ^a^	1.39 ± 0.05 ^b^
**4 HBA**	16.30 + 1.34 ^a^	4.29 + 0.53 ^b^	ND	ND	ND	ND
**ChA**	45.59 ± 3.50 ^a^	15.33 ± 3.87 ^b^	170.61 ± 5.12 ^a^	50.21 ± 5.17 ^b^	70.77 ± 3.63 ^a^	20.51 ± 1.45 ^b^
**pCA**	12.06 ± 1.22 ^a^	ND	60.42 ± 3.65 ^a^	ND	70.70 ± 2.63 ^a^	ND
Flavonoids						
**Cat**	22.11 ± 1.51 ^a^	10.02 ± 6.45 ^b^	20.22 ± 1.46 ^a^	ND	10.17 ± 0.39 ^a^	ND
**ECat**	21.19 ± 1.98 ^a^	14.07 ± 7.88 ^b^	3.06 ± 0.21 ^a^	ND	2.88 ± 0.01 ^a^	ND
**K 3 gluc**	30.93 ± 1.33 ^a^	ND	140.90 ± 4.61 ^a^	3.23 ± 0.11 ^b^	110.05 ± 9.01 ^a^	4.43 ± 0.61 ^b^
**Myrc**	98.12 + 4.78 ^a^	ND	ND	ND	ND	ND
**Quer**	3.30 + 0.11 ^a^	ND	ND	ND	ND	ND
Anthocyanins						
**Delp 3 gluc**	80.58 ± 6.21 ^a^	1.73 ± 0.01 ^b^	1100.56 ± 14.01 ^a^	30.79 ± 2.01 ^b^	809.26 ± 7.01	ND
**Cy 3 gluc**	26.10 ± 1.33 ^a^	2.62 ± 1.66 ^b^	400.77 ± 9.52 ^a^	45.89 ± 3.49 ^b^	3.41 ± 0.22	ND
**PT 3 glu**	63.71 ± 5.12 ^a^	4.55 ± 2.01 ^b^	2.99 ± 0.11 ^a^	170.71 ± 11.03 ^b^	2100.20 ± 10.46 ^a^	90.8 ± 4.51 ^b^
**Malv 3,5 di**	500.28 + 4.98 ^a^	130.54 ± 8.52 ^b^	140.17 ± 5.77 ^a^	39.18 ± 2.44 ^b^	33.88 ± 1.71 ^a^	4.05 + 0.12 ^b^
**Malv 3 gal**	25.84 + 1.49	ND	190.33 + 6.33	ND	46.26 ± 2.51	ND
**Malv 3 glu**	40.02 ± 2.29 ^a^	3.37 ± 1.66 ^b^	3100.22 ± 9.01 ^a^	250.64 ± 6.60 ^b^	730.19 ± 5.46 ^a^	65.8 ± 0.95 ^b^

Data are expressed as mean ± standard deviation (*n* = 3). Different lowercase letters for each variety within a row indicate a significant difference by cooking (*p* < 0.05). dw; dry weight. GA: gallic acid; SA: sinapic acid; 4 HBA: 4-hydroxybenzoic acid; FA: ferulic acid; ChA: chlorogenic acid; pCA: p-coumaric acid; PA: protocatechuic acid; Cat: catechin; ECat: epicatechin; K 3 gluc: kaempferol 3-glucoside; Myrc: myricetin; Querc: quercetin; Delp 3 gluc: delphinidin 3-glucoside; Cy 3 gluc: cyanidin 3-glucoside, PT 3 glu: petunidin-3-glucoside; Malv 3,5 di: malvin 3,5 diglucoside; Malv 3 gal: malvidin-3 galactoside; Malv 3 glu: malvidin-3-glucoside; ND: not detected.

## Data Availability

Data are contained within the article.
